# Emergent Median Sternotomy for Mediastinal Hematoma: A Rare Complication following Internal Jugular Vein Catheterization for Chemoport Insertion—A Case Report and Review of Relevant Literature

**DOI:** 10.1155/2014/190172

**Published:** 2014-01-30

**Authors:** Saptarshi Biswas, Marwa Sidani, Sunil Abrol

**Affiliations:** Department of Surgery, Brookdale University Hospital Medical Center, Brooklyn, NY 11212, USA

## Abstract

Mediastinal hematoma is a rare complication following insertion of a central venous catheter with only few cases reported in the English literature. We report a case of a 71-year-old female who was admitted for elective chemoport placement. USG guided right internal jugular access was attempted using the Seldinger technique. Resistance was met while threading the guidewire. USG showed a chronic clot burden in the RIJ. A microvascular access was established under fluoroscopic guidance. Rest of the procedure was completed without any further issues. Following extubation, the patient complained of right-sided chest pain radiating to the back. Chest X-ray revealed a contained white out in the right upper lung field. She became hemodynamically unstable. Repeated X-ray showed progression of the hematoma. Median Sternotomy showed posterior mediastinal hematoma tracking into right pleural cavity. Active bleeding from the puncture site at RIJ-SCL junction was repaired. Patient had an uneventful recovery. Injury to the central venous system is the result of either penetrating trauma or iatrogenic causes as in our case. A possible explanation of our complication may be attributed to the forced manipulation of the dilator or guidewire against resistance. Clavicle and sternum offer bony protection to the underlying vital venous structures and injuries often need sternotomy with or without neck extension. Division of the clavicle and disarticulation of the sternoclavicular joint may be required for optimum exposure. Meticulous surgical technique, knowledge of the possible complications, and close monitoring in the postprocedural period are of utmost importance. Chest X-ray showed to be routinely done to detect any complication early.

## 1. Introduction

Internal jugular vein catheterization is a fairly common procedure for inserting a chemotherapy port. In fact, more than 5 million central venous catheters are inserted every year in the United States [1]. However, such catheterization may be associated with serious life threatening complications, which have been reported to occur in 6.2–10.7% [2] of patients. Various complications can be encountered including a higher risk of pneumothorax, puncture of the carotid or subclavian artery, cardiac tamponade, or hemothorax. Other less serious risk factors include infection, thrombosis, or factors related to maintenance of the central line.

In this paper we present a case report of a relatively rare complication of mediastinal hematoma following internal jugular vein catheterization for chemoport insertion. We also discuss the relevant literature regarding complications associated with central line placement as well as the various available treatment options.

## 2. Case Report

A 71-year-old female was admitted under the surgical service for elective chemoport placement. She was known diabetic and hypertensive and well controlled on medications. She was also diagnosed with stage II gastric cancer. She underwent distal gastrectomy and Bilroth II reconstruction several months ago and was scheduled for chemotherapy as part of her management.

Just prior to the procedure the patient developed symptomatic bradycardia manifested as near syncope with her heart rate dropping to 40 beats per minute. Consequently, the procedure was cancelled and the patient was admitted to the medical service for further workup. Cardiology was consulted and she was started on Plavix in addition to the Lovenox she was having for DVT prophylaxis. Workup including transthoracic echocardiography (TTE) was within normal and she was cleared for the procedure. Later Plavix was stopped at the time of the procedure.

**Figure 1:**
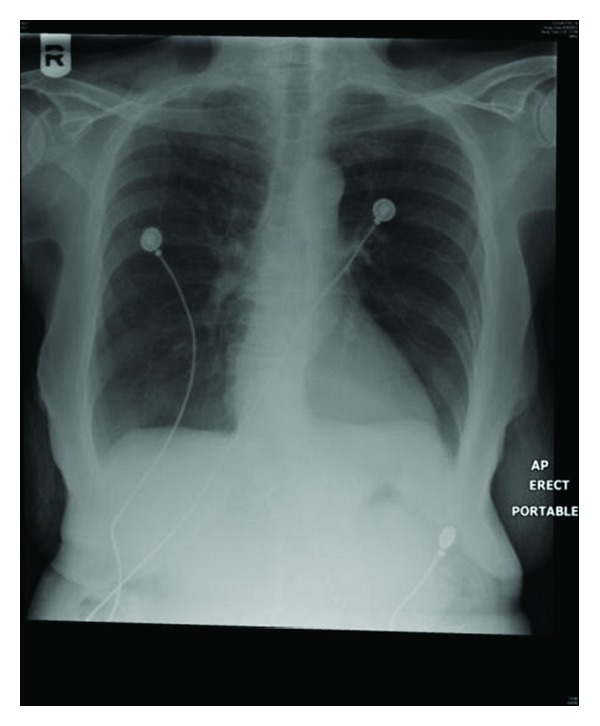
Preoperative chest X-ray: essentially normal.

**Figure 2:**
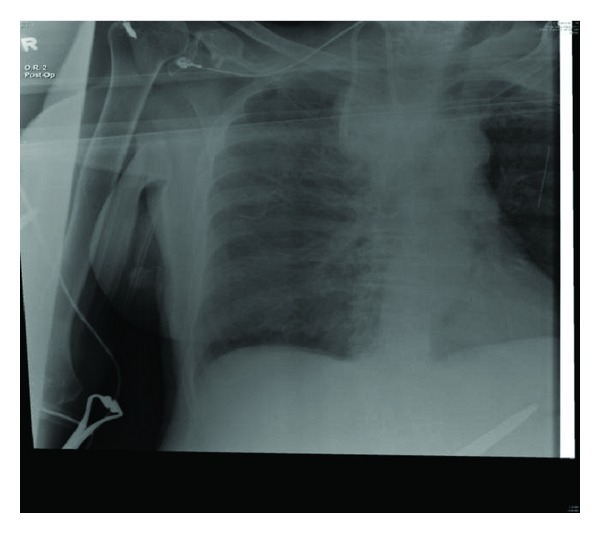
Chest X-ray showing right upper lobe whitening.

The patient was taken to operating room the following day for the placement of a chemoport. Her chest X-ray (Figure 1) was normal and she was asymptomatic. General anesthesia was induced with no complications. The patient was draped and positioned in Trendelenberg. Ultrasound guided insertion of a right internal jugular venous (IJV) access was attempted with a total of 4 trials using Seldinger's technique. Resistance was met while threading the guide wire. Ultrasound examination showed a chronic clot burden in the Right IJV. The vascular surgery team was consulted intra operatively. A right IJV access using a microset access under fluoroscopic guidance was obtained. The procedure was then completed without any further issues. Following extubation, and upon transfer of the patient to the stretcher, she started complaining of right-sided chest pain radiating to the back. She became tachycardic with a heart rate ranging between 105 and 110 beats per minute. CXR was immediately obtained and it revealed a contained white out in the right upper lung field (Figure 2).

**Figure 3:**
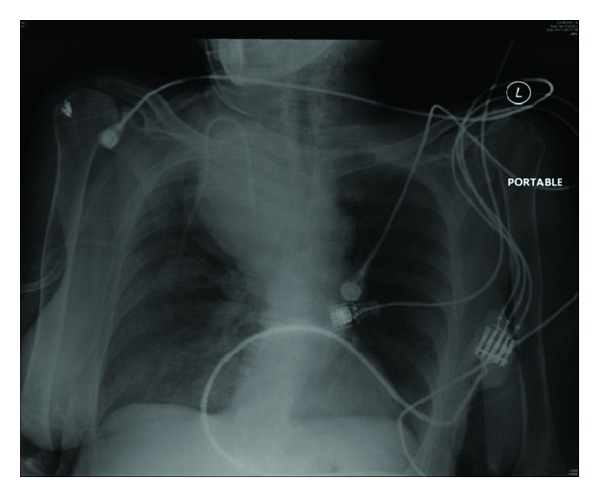
Postprocedural chest X-ray showing right mediastinal hematoma. Thoracic and vascular surgery team was consulted immediately and the decision was made to emergently take the patient to the operating room for evacuation of a mediastinal hematoma. Central and arterial access was obtained and transfusion of packed red blood cells (PRBC) was started. The hemodynamic status improved.

Soon after, the patient decompensated with the systolic blood pressure dropping to 60 mm Hg. Volume resuscitation with crystalloids was started and the patient was cross-matched for transfusion of blood products. A third CXR was obtained during this time revealing the progression of a hemothorax in the right side of the chest (Figure 3).

**Figure 4:**
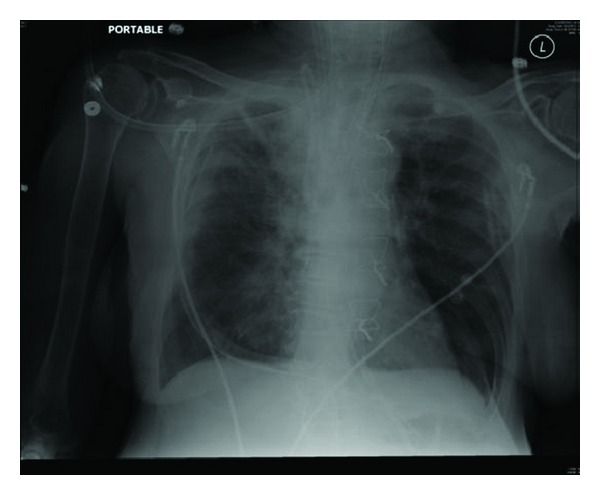
Postoperative chest X-ray: significant improvement of the right mediastinal hemothorax.

A median sternotomy was performed. Upon exploration of the thorax, a posterior mediastinal hematoma tracking into right pleural cavity was detected. There was active bleeding from the puncture site at IJV-subclavian (SCV) junction. By the end of the surgery, the patient received a total of four units of (PRBC), two units of fresh frozen plasma (FFP), and one unit of platelets. The patient was transferred to the surgical intensive care unit (SICU) postoperatively intubated and hemodynamically stable. A right pleural chest tube was kept in place for drainage. Postoperative CXR was done showing marked improvement of the right-sided hemothorax (Figure 4). The next morning, the patient was extubated and the mediastinal chest tube was removed. The patient had an unremarkable recovery course and was transferred out of the SICU after removal of the right pleural chest tube.

## 3. Discussion

Injury to the central venous system in most cases is the result of either penetrating trauma, like gunshot wounds, or iatrogenic causes like central line catheter placement. Penetrating injuries especially secondary to gunshot wounds to the subclavian and innominate veins can often result in rapid exsanguination resulting in significant mortality [3–7] in this patient group.

Right internal jugular vein (RIJV) is often selected as an ideal vein for central venous access for chemoport access because of its straight course, reduced risk of malposition, and thrombosis. However, mechanical complications from central venous catheter insertion are reported to occur in 5 to 19% of patients, infectious complications in 5 to 26%, and thrombotic complications in 2 to 26% [1].

Central venous catheterizations along with erosions are more common causes of injury to the innominate-superior vena cava (SVC) confluence compared to penetrating trauma [3, 8–22]. However, such complications of catheter placement are relatively rare compared to the more common complications like pneumothorax, infection, and intravascular thrombosis [3].

The innominate-SVC junction is in close proximity to the pleural and pericardial spaces. Localized central line perforation within the mediastinal pleura may not cause significant complications if identified early and the catheter removed promptly. However, if the perforation is delayed or missed, hydrothorax communicates to the pleural space. The hydrothorax is usually on the contralateral side of the chest from the central line placement. In some cases, it may be bilateral [22]. Acute perforation of the innominate-SVC junction can result in cardiac tamponade resulting in sudden hemodynamic decompensation [3, 15–17].

Elderly, bed-ridden, debilitated, and malnourished patients with chronic underlying disease processes are predisposed to chronic catheter injury at the innominate-caval junction. Chronic catheter injuries are mostly associated with left sided catheter insertion [22] with the central tip eroding the cephalad portion of SVC. The probable explanation is that the anatomy of the left innominate vein is relatively more horizontal than the right innominate vein and the junction with the SVC is almost at a right angle. Thus, catheter tip inserted through the left side is more likely to impinge on the right caval wall. Abnormal angulation of the catheter relative to the SVC, infusion of hyperosmolar solution and undue flexion, and extension of the patient's neck can result in erosion. Delayed perforation usually results in unilateral right side pleural effusion. However it can be bilateral in 33% of cases [22].

Internal jugular vein central line insertion is widely used as a way for venous access for chemotherapy port. The occurrence of mediastinal hematoma resulting from vascular injury in close proximity to the mediastinum and the need for emergent thoracotomy due to hemodynamic instability is an extremely rare complication. It is documented in the literature in very few case reports [23]. Gupta et al. [23] in a case report published in 2011 mentioned 8 cases of mediastinal hematoma following central line placement.

Arik et al. [24] reported a case of mediastinal hematoma after the insertion of a left subclavian venous catheter in a patient with end stage renal failure for the purpose of hemodialysis. The guide wire most likely penetrated the subclavian vein and caused bleeding that resulted in mediastinal hematoma and eventual death of the patient.

A possible explanation of the mechanism of vascular complication includes forced manipulation of the dilators or guide wires against resistance. In our case, we encountered appreciable resistance while threading the guidewire when it reached the IJ junction. We were able to recognize the difficulty of threading the guidewire into the IJ and to identify a chronic clot burden in the right IJ by using ultrasound examination. We attributed the resistance we met to a thrombus at the IJ junction, although that is considered to be a rare incidence.

Gupta et al. [23] presented a case report of a mediastinal hematoma in a 33-year-old male after placement of a right subclavian central line for intra and postoperative central venous pressure monitoring during a renal transplantation surgery. The mediastinal hematoma was discovered after routine CXR done postoperatively. The right mediastinal hematoma was managed conservatively with repeated chest radiographs. In their discussion, Gupta et al. presented 8 cases of occurrence of mediastinal hematoma after insertion of a central line. Only one case, reported by Doi et al. [25], required a thoracotomy. Three cases presented required coil embolization of the internal mammary artery, two cases were managed with insertion of bilateral intercostal drains for hydrothorax, and two cases were managed conservatively.

Doi et al. [25] described a posterior mediastinal hematoma that developed after right internal jugular cannulation for central venous and PA catheter in a patient undergoing cardiac surgery. The patient experienced hemodynamic instability and stridor on the day following the operation due to tracheal compression by the hematoma. It was speculated to be due to a misplacement of either the internal jugular catheter or the guide wire into the azygous vein. The hematoma was evacuated with a thoracotomy.

Naguib et al. [26] described a case of hydromediastinum and bilateral hydrothorax after a subclavian line insertion in a 28-year old male after multiple fractures due to motor vehicle accident. The left subclavian vein was cannulated intraoperatively. Postoperative CXR was done and was negative for pneumo- or hemothorax. However, three hours later, the patient became hemodynamically unstable, which required further CXR revealing widening of the mediastinum. The patient was managed with bilateral chest tubes for bilateral hydrothorax and the hydromediastinum was managed conservatively.

Also, Chemelli et al. [27] conducted a retrospective analysis of five patients with iatrogenic arterial lesions of the internal mammary artery (IMA). The lesions occurred in three patients from a puncture of the subclavian vein during insertion of a central venous catheter and in two patients from a puncture of the subclavian vein for insertion of a pacemaker lead. Microcoil embolization was performed to control the source of bleeding.

Hohlrieder et al. [28] reported a life-threatening mediastinal hematoma in a 6-month-old girl during surgical correction of scaphocephaly. The left subclavian vein was successfully punctured on the first attempt using the Seldinger technique. The patient showed persistent hemodynamic instability requiring the use of emergent transesophageal echocardiography (TEE) for further investigation. A large mediastinal hematoma compressing the right atrium and the SVC was detected due to dislocation of the left subclavian catheter. The cannula was removed and the patient was managed conservatively.

Innominate-caval injuries are usually related to catheter placement and can result from central line placement at either side. Acute injuries can often cause cardiac tamponade. For innominate-caval confluence exposure, median sternotomy alone is adequate. However, extension of the incision into the neck and resection of the clavicle are necessary for adequate exposure of the subclavian Internal jugular junction. Clamps, ligations or cardiopulmonary bypass can achieve vascular control. Asymptomatic mediastinal hematoma can sometimes be observed and managed conservatively [3].

Moreover, the clavicle as well as the sternum offer bony protection to the vital central venous system. Although some subclavian venous injuries can be managed using only division of the clavicle and disarticulation of the sternoclavicular joint, optimum exposure is achieved when median sternotomy is performed. Thoracotomy does not provide a good exposure of the subclavian-IJ junction. Because of the difficulty of vascular reconstruction at venous confluences some authors recommend simple ligation of the venous injuries at the subclavian-IJ junction. Others like Baumgartner et al. [3], however, prefer primary repair compared to ligation in their series.

## 4. Conclusion 

Meticulous surgical technique, knowledge of the possible complications, and close monitoring of the patient in the perioperative period are required in the management of central line, dialysis catheter, and chemoport placements.

Postprocedural chest radiographs are useful in detecting complications early as in our case and should be done routinely.

## References

[1] Raad I (1998). Intravascular-catheter-related infections. *The Lancet*.

[2] McGee DC, Gould MK (2003). Preventing complications of central venous catheterization. *The New England Journal of Medicine*.

[3] Baumgartner FJ, Rayhanabad J, Bongard FS, Milliken JC, Donayre C, Klein SR (1999). Central venous injuries of the subclavian-jugular and innominate-caval confluences. *Texas Heart Institute Journal*.

[4] Rao PM, Ivatury RR, Sharma P, Vinzons AT, Nassoura Z, Stahl WM (1993). Cervical vascular injuries: a trauma center experience. *Surgery*.

[5] Degiannis E, Velmahos G, Krawczykowski D, Levy RD, Souter I, Saadia R (1994). Penetrating injuries of the subclavian vessels. *British Journal of Surgery*.

[6] Demetriades D, Rabinowitz B, Pezikis A, Franklin J, Palexas G (1987). Subclavian vascular injuries. *British Journal of Surgery*.

[7] Robbs JV, Reddy E (1987). Management options for penetrating injuries to the great veins of the neck and superior mediastinum. *Surgery Gynecology and Obstetrics*.

[8] Duntley P, Siever J, Korwes ML, Harpel K, Heffner JE (1992). Vascular erosion by central venous catheters: clinical features and outcome. *Chest*.

[9] Ellis LM, Vogel SB, Copeland EM (1989). Central venous catheter vascular erosions. Diagnosis and clinical course. *Annals of Surgery*.

[10] Iberti TJ, Katz LB, Reiner MA (1983). Hydrothorax as a late complication of central venous indwelling catheters. *Surgery*.

[11] Chute E, Cerra FB (1982). Late development of hydrothorax and hydromediastinum in patients with central venous catheters. *Critical Care Medicine*.

[12] Dailey RH (1988). Late vascular perforations by CVP catheter tips. *Journal of Emergency Medicine*.

[13] Kapadia CB, Heard SO, Yeston NS (1988). Delayed recognition of vascular complications caused by central venous catheters. *Journal of Clinical Monitoring*.

[14] Mukau L, Talamini MA, Sitzmann JV (1991). Risk factors for central venous catheter-related vascular erosions. *Journal of Parenteral and Enteral Nutrition*.

[15] Chabanier A, Dany F, Brutus P, Vergnoux H (1988). Iatrogenic cardiac tamponade after central venous catheter. *Clinical Cardiology*.

[16] Barton BR, Hermann G, Weil R (1983). Cardiothoracic emergencies associated with subclavian hemodialysis catheters. *Journal of the American Medical Association*.

[17] Fischer GW, Scherz RG (1973). Neck vein catheters and pericardial tamponade. *Pediatrics*.

[18] Armstrong CW, Mayhall CG (1983). Contralateral hydrothorax following subclavian catheter replacement using a guidewire. *Chest*.

[19] McDonnell PJ, Qualman SJ, Hutchins GM (1984). Bilateral hydrothorax as a life-threatening complication of central venous hyperalimentation. *Surgery Gynecology and Obstetrics*.

[20] Agarwal MK, Banner AS, Addington WW (1978). Bilateral hydrothorax from unilateral subclavian vein catheterization. *Journal of the American Medical Association*.

[21] Flatley ME, Schapira RM (1993). Hydropneumomediastinum and bilateral hydropneumothorax as delayed complications of central venous catheterization. *Chest*.

[22] Li PK, Taylor CW, Chung RS (1997). Delayed hydrothorax: a complication of central venous catheterization. *Surgical Rounds*.

[23] Gupta P, Guleria S, Sharma S (2011). Mediastinal haematoma: a rare complication following insertion of central venous catheter. *The Indian Journal of Chest Diseases & Allied Sciences*.

[24] Arik N, Akpolat T, Demirkan F (1993). Mediastinal hematoma: a rare complication of subclavian catheterization for hemodialysis. *Nephron*.

[25] Doi A, Iida H, Saitoh Y, Sunazawa T, Tajika Y (2009). A posterior mediastinal hematoma causing tracheal obstruction after internal jugular cannulation for cardiac surgery. *Journal of Cardiothoracic and Vascular Anesthesia*.

[26] Naguib M, Farag H, Joshi RN (1985). Bilateral hydrothorax and hydromediastinum after a subclavian line insertion. *Canadian Anaesthetists Society Journal*.

[27] Chemelli AP, Chemelli-Steingruber IE, Bonaros N (2009). Coil embolization of internal mammary artery injured during central vein catheter and cardiac pacemaker lead insertion. *European Journal of Radiology*.

[28] Hohlrieder M, Oberhammer R, Lorenz IH, Margreiter J, Kühbacher G, Keller C (2004). Life-threatening mediastinal hematoma caused by extravascular infusion through a triple-lumen central venous catheter. *Anesthesia and Analgesia*.

